# Supporting the improvement and management of prescribing for urinary tract infections (SIMPle): protocol for a cluster randomized trial

**DOI:** 10.1186/1745-6215-14-441

**Published:** 2013-12-23

**Authors:** Sinead Duane, Aoife Callan, Sandra Galvin, Andrew W Murphy, Christine Domegan, Eamon O’Shea, Martin Cormican, Kathleen Bennett, Martin O’Donnell, Akke Vellinga

**Affiliations:** 1 Discipline of General Practice, School of Medicine, National University of Ireland, Galway, Ireland; 2 Discipline of Economics, JE Cairnes School of Business and Economics, National University of Ireland, Galway, Ireland; 3 Irish Centre for Social Gerontology, National University of Ireland, Galway, Ireland; 4 Department of Marketing, JE Cairnes School of Business and Economics, National University of Ireland, Galway, Ireland; 5 Discipline of Bacteriology, School of Medicine, National University of Ireland, Galway, Ireland; 6 Department of Medical Microbiology, University Hospital Galway, Galway, Ireland; 7 Department of Pharmacology & Therapeutics, Trinity College Dublin, Dublin, Ireland; 8 Health Research Board Clinical Research Facility Galway, National University of Ireland, Galway, Ireland

**Keywords:** Antimicrobial, Intervention, Prescribing, Primary care, Social marketing, Urinary tract infection

## Abstract

**Background:**

The overuse of antimicrobials is recognized as the main selective pressure driving the emergence and spread of antimicrobial resistance in human bacterial pathogens. Urinary tract infections (UTIs) are among the most common infections presented in primary care and empirical antimicrobial treatment is currently recommended. Previous research has identified that a substantial proportion of Irish general practitioners (GPs) prescribe antimicrobials for UTIs that are not in accordance with the Guidelines for Antimicrobial Prescribing in Primary Care in Ireland. The aim of this trial is to design, implement and evaluate the effectiveness of a complex intervention on GP antimicrobial prescribing and adult (18 years of age and over) patients’ antimicrobial consumption when presenting with a suspected UTI.

**Methods/design:**

The Supporting the Improvement and Management of Prescribing for urinary tract infections (SIMPle) study is a three-armed intervention with practice-level randomization. Adult patients presenting with suspected UTIs in primary care will be included in the study.

The intervention integrates components for both GPs and patients. For GPs the intervention includes interactive workshops, audit and feedback reports and automated electronic prompts summarizing recommended first-line antimicrobial treatment and, for one intervention arm, a recommendation to consider delayed antimicrobial treatment. For patients, multimedia applications and information leaflets are included. Thirty practices will be recruited to the study; laboratory data indicate that 2,038 patients will be prescribed an antimicrobial in the study. The primary outcome is a change in prescribing of first-line antimicrobials for UTIs in accordance with the Guidelines for Antimicrobial Prescribing in Primary Care in Ireland. The study will take place over 15 months with a six-month intervention period. Data will be collected through a remote electronic anonymized data-extraction system, a text-messaging system and GP and patient interviews and surveys. The intervention will be strengthened by the implementation of a social marketing framework and an economic evaluation.

**Trial registration:**

This intervention is registered at ClinicalTrials.gov, ID NCT01913860.

## Background

Antimicrobial resistance (AMR) is an important and complex public health problem [1]. The spread of AMR has led to the increased use of reserved antimicrobial agents [2] in an era where few new antimicrobial agents are in production [3].

Today, 80% of antimicrobial prescribing takes place in the community by general practitioners (GPs) [4]. Ireland is one of only three countries in Europe where the level of outpatient antimicrobial prescribing is increasing [5,6]. Within this context, the inappropriate and over-prescription of antimicrobials by GPs is a recognized factor contributing to the spread of AMR [7-10]. The Guidelines for Antimicrobial Prescribing in Primary Care in Ireland [11] provide advice on the selection of antimicrobial drugs for common infections and recommend the use of specific antimicrobials, with reserved drugs for more serious infections. However, despite the widespread availability of these guidelines, recent research has identified that less than 40% of outpatient prescriptions for urinary tract infections (UTIs) are made out according to first-line recommendations [12].

Urinary tract infections are predominantly caused by a bacteria; *Escherichia coli*[6,13,14], and are generally treated empirically, prior to the results of antimicrobial susceptibility testing [11,12,15]. Antimicrobial resistance is now a critical factor in the treatment of UTIs [9,12,16], the second most common bacterial infection in primary care [6,12,13,15,17].

### Changing prescribing and consumption antimicrobial behaviours

Antimicrobial resistance is complex [7,18], dynamic [19] and continuous [20], meaning that no single solution will manage the problem effectively. Multifaceted interventions aimed at multiple stakeholders (GP, patients and the wider community) have been shown to be successful in reducing inappropriate prescribing [2,21-23] and can bring about social change by addressing local barriers to change [24]. Patients may have preconceived expectations of the consultation [23], determined by their prior experience within the practice and the treatment of a recurring condition in some cases [25]. However, a review of patient-orientated interventions to improve antimicrobial prescribing concluded that change is better achieved by encouraging health professionals rather than by educating patients about the negative aspects of antimicrobials [2]. The GP’s decision to prescribe antimicrobials should be a balance between the treatment of the individual in the short term and its harmful impact on society in the long term [19,26]. General practitioners prescribe antimicrobials to treat (bacterial) infection, to guard against the risk of a missed diagnosis [19], or because they believe the patient expects this outcome from the consultation [25]; therefore, to obtain a behavioural change, many factors need to be addressed [27].

Educational interventions aimed at the prescriber, the GP, have shown some successes. Improvements in overall prescribing practices in primary care have been linked with the use of small interactive workshops with health care professionals, which provide a greater change in prescribing behaviours when compared with a passive lecture-style format [28-31]. An interactive workshop style is more likely to identify multifactorial causes in inappropriate prescribing, leading to the provision of tailored behavioural change methods for GPs [2]. Electronic prescribing prompts have successfully increased GP adherence to prescribing guidelines for a range of common illnesses [32-34]. Electronic prompts have also been successful in increasing the use of specific antimicrobial drug choices, such as first-line antimicrobial treatment for common infections [35]. Currently, prescribing prompts are not commonly available within GP patient management software systems in Ireland.

The use of audit and feedback of information in conjunction with other intervention methods (delayed prescribing, educational material or electronic prompts) has also proven effective in improving GPs antimicrobial prescribing behaviours [2,36]. General practitioners in the UK receive routine feedback on their prescribing practices and are among the lowest community prescribers of antimicrobials in Europe [30,37]. Currently, Irish GPs cannot readily access information on their prescribing practices, despite evidence to suggest that feedback can successfully reduce antimicrobial prescribing in the Irish primary care setting [36].

The use of delayed antimicrobial prescribing for viral infections in primary care in the UK has been credited with achieving a 50% reduction in antimicrobial use [22,38,39]. Empirical antimicrobial treatment for UTIs is currently recommended in the Guidelines for Antimicrobial Prescribing in Primary Care in Ireland [11]. However, in at least 50% of patients with UTIs, an antimicrobial may not be required, as the infection resolves naturally [40-42]. Qualitative studies investigating attitudes to delayed antimicrobial prescribing in primary care have indicated that both patients and GPs are satisfied with this treatment format and welcome this 'safety net’ approach as a feasible treatment strategy [23,43-45].

Between the GP and patient, the communication of such treatment strategies as delayed prescribing can be viewed as part of a shared decision-making process [23]. A shared decision-making process can empower patients through a greater understanding of the issues involved [46,47]. Shared decision making with delayed prescribing allows patients to prioritize what they value most; increasing their chances of a quick recovery or reducing their chances of side effects and reconsultations in the future [47,48].

The leading systematic review in the area of complex prescribing interventions in primary care called for innovative intervention methods to be developed [2]. Around 75% of a general practice’s registered patients will wait in the practice waiting room each year and the demand for easy-to-understand healthcare information is increasing [49]. The use of informative material in GPs waiting rooms, such as educational videos and interactive games, to create awareness and explain the problems associated with the overuse of antimicrobials in primary care, has also been recommended through the European Antibiotics Awareness Day [50].

Educational videos displayed in the practice waiting room can also increase patients’ understanding and satisfaction of their care, as well as empowering patients to discuss their treatments further with the GP [51,52]. Previous research examined the effectiveness of a multimedia campaign in comparison with a static educational brochure to improve treatment for chronic illness in practice waiting rooms with patients with low health literacy [53,54]. This approach was considered novel, effective and acceptable in improving health care management, by empowering patients to discuss making positive changes to their treatment with their GP [54]. Audiovisual messages played in practice waiting rooms have also proven effective in increasing patient uptake of vaccines [55].

Mobile phone technology can facilitate rapid and cost-effective access to a study group of interest to facilitate data collection. Previous programmes have successfully used text messages to assess consumption and adherence to antimicrobial treatments with 72% patient participation for follow-up [56].

### Intervention development overview

Social marketing is the conceptual framework that guided the development of this intervention, by integrating knowledge from such disciplines as psychology, anthropology and sociology [57-59] with commercial marketing techniques [59-63]. Social marketing interventions address three key areas: understanding current behaviours; identifying determinants and identifying mechanisms for change [64].

Owing to the intricate factors that influence the decision to prescribe an antimicrobial, not all strategies will work with all GPs in all regions [2]. The SIMPle study focuses on behavioural changes that are feasible and self-sustaining, given the available resources. The appropriateness of changes and their feasibility within the GP practice setting were also considered [65].

Formative (qualitative) research explored the culture of antimicrobial prescribing from both the GP’s and the patient’s perspective. Through a series of interviews with GPs (*n* = 16) and focus groups with patients (*n* = 35), the predictors of a GP’s decision to prescribe an antimicrobial and the patient’s expectation to receive an antimicrobial were explored. The questions were guided by a combination of theoretical frameworks, the transtheoretical model [66] and the buyer behaviour decision-making model [67], which together explored six key areas: the stage of change, consequences, trade-offs, other influences, segmentation and competition [68].

As a result of this formative research process, five core outcomes were achieved:

1. The expectations of the patient relating to the UTI consultation with GPs were characterized.

2. The factors that impact the GPs decision to prescribe an antimicrobial were defined.

3. The key messages central to the design and development of this intervention were specified for both the GP and the patient.

4. The results supported the development of quantitative evaluation components, such as a baseline questionnaire to monitor changes in knowledge, attitudes and awareness for both GP and patient.

5. The behavioural theoretical framework underpinning the design and development of this complex intervention was defined.

The multiple interactive components of this intervention were informed by both the peer-reviewed literature and formative research. In short, the SIMPle study will integrate:

• A professional development programme for the GP, which includes interactive workshops, audit and feedback reports on antimicrobial prescribing and electronic antimicrobial prescribing prompts to improve the quality of prescribing. The quality of prescribing is defined within this study as the prospective prescribing of first-line antimicrobials in accordance with the Guidelines for Antimicrobial Prescribing in Primary Care in Ireland.

• Delayed antimicrobial prescribing for UTIs in one study arm, to decrease the consumption of antimicrobials.

• A supportive framework to inform patients of AMR through multimedia applications within the waiting room of the GP.

• Novel e-health technology, which includes an electronic data extraction system that will remotely collect anonymized data from all consultations with patients diagnosed with a UTI by the GPs and mobile health technology to monitor and record antimicrobial consumption behaviour of patients.

By integrating this intervention into routine care and making all material freely available at the end of the intervention, the SIMPle study strives to be sustainable and self-promoting and, thereby, implemented in primary care in Ireland beyond the intervention period.

### Aim and objectives

#### Aim of the SIMPle study

To design, implement and evaluate the effectiveness of a complex intervention on GP antimicrobial prescribing and adult (18 years of age and over) patients’ antimicrobial consumption when presenting with a suspected UTI.

#### Primary objective

To increase the number of first-line antimicrobial prescriptions, as recommended in the Guidelines for Antimicrobial Prescribing in Primary Care in Ireland (2011), for suspected UTIs in primary care by 10% in adult patients.

#### Secondary objectives

1. To compare the effect of the intervention on the frequency of antimicrobial prescribing and antimicrobial consumption in patients presenting with a UTI.

2. To measure the uptake and impact of delayed antimicrobial prescribing for UTIs and the impact of this treatment approach on UTI GP reconsultation visits.

3. To assess a change in cognitive beliefs (knowledge and attitudes) of GPs related to antimicrobial prescribing.

4. To conduct a cost-effectiveness and process evaluation of the SIMPle intervention.

5. To compare the prescribing rates of the intervention arms with regional UTI antimicrobial prescribing rates.

## Methods and design

### Setting

The cluster for this intervention is the practice, and all GPs within each practice will be invited to participate. The baseline population for recruitment of patients will be formed by all practices in the West of Ireland who submit urine samples to the Galway University Hospitals (GUH) laboratory. The most popular patient management software system was chosen, because the SIMPle study builds on remote data extraction, the provision of audit and feedback reports and computer prompts integrated within the GP’s patient management software system. If successful, the intervention could be integrated within the other patient management software systems, of which there are five. In summary, practice eligibility criteria are such that all practices must have the specified patient management software system and submit urine samples to the GUH laboratory.

### Study design

**Figure 1:**
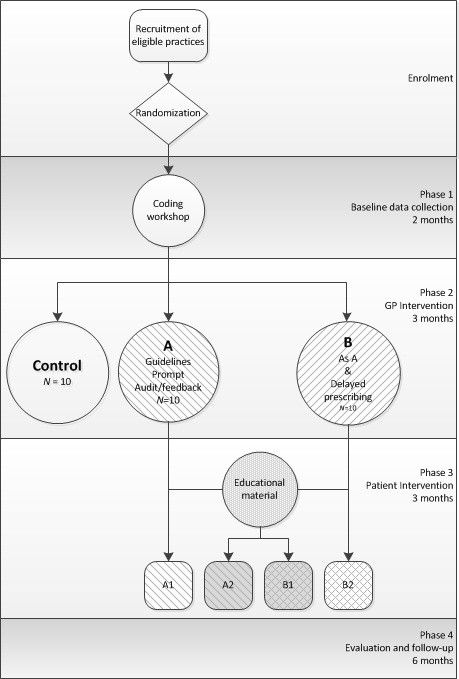
Study design.

This study will be undertaken in four phases: Phase 1, baseline data collection; Phase 2, GP intervention; Phase 3, patient intervention and Phase 4, endpoint data collection. The study design is summarized in Figure 1.

### Implementation

Computerized remote data extraction is facilitated by the Irish Primary Care Research Network (iPCRN) [69] and patient data are identified through the appropriate coding of suspected UTI consultations in the patient management software system. To promote and encourage consultation coding, which is currently not routine practice, the intervention will be preceded by a coding workshop at the beginning of Phase 1. All practices will be required to register with the iPCRN during or before the coding workshop. Practices will be monitored for two months after the delivery of the workshop to establish uptake of coding, whilst also facilitating a baseline data collection period (Phase 1). Phase 2 will begin with an interactive workshop (intervention arms A and B), which will introduce the intervention components specific to each arm. Phase 3 will see the roll-out of the patient education in all of the practices. The antimicrobial prescribing within each practice will be monitored for six months during Phases 2 and 3 through audit and feedback reports. Evaluation of the impact of the intervention will be carried out in Phase 4.

### Practice identification and randomization

A list of all practices submitting urine samples to GUH was obtained and practices were contacted by phone to establish what patient management software system they used. Practices using the selected patient management software system were thereby identified. The first 30 practices were invited to participate by an invitation letter, which provided an overview of the study, benefits for participation and the contact details of the research team.

Within 10 days of receipt of this invitation, practices were telephoned to determine their interest and willingness to participate in the study. If a practice declined to participate, the reason for not agreeing was recorded and that practice was replaced by another from the eligibility list. This process continued until 30 practices were enrolled. Practices were then randomized to intervention and control arms, using a list of computer-generated numbers. The workshop facilitators were blinded to the allocation throughout the delivery of the coding workshops in Phase 1.

#### Patient eligibility

All patients are eligible if aged 18 years and over and presenting with symptoms of an UTI, as determined by the GP through the consultation coding.

### Patient enrolment

All patients consulting in the participating practices will be informed of the study through information posters in the waiting area from Phase 1.

In Phase 2, GPs will be encouraged to submit a urine sample from each patient presenting with a suspected UTI to the GUH laboratory and request the patient’s mobile telephone number during the consultation. The patient’s mobile telephone number will be recorded on the laboratory diagnostic request form submitted with the urine sample to the GUH laboratory. Mobile telephone numbers will be collected on a daily basis from this laboratory. Each mobile telephone number will be entered into a computer program, which will generate automated text messages. The first text message will confirm consent.

### Sample size

#### Primary outcome: adherence to the guidelines for antimicrobial prescribing in primary care in Ireland (control - intervention arm a)

Previous research has established that 56% of UTI patients receive an antimicrobial, with only 38% of prescriptions made out for the recommended first-line treatment [8]. Sample size calculations are based on an absolute 10% increase in first-line prescriptions according to guidelines (primary outcome).

Additional assumptions for sample size calculations are:

• Power of 80% and *α* of 5%.

• Practice attrition is dependent on the completeness of consultation coding, which will be monitored and corrected during Phase 1.

• Patient attrition will be close to 0%, as all coded consultations will be included.

• Improvement in the control group due to participation in the coding workshops has been anticipated.

• An intracluster correlation (ICC) of 1% was obtained from a previous study in this area [6,12].

A total sample size of 920 patients recruited from 20 practices gives a power of 80% to detect a significant change in the proportion of patients to receive first-line antimicrobial when treated for UTI when the ICC is 1%.

### Feasibility

According to the most recent laboratory urine sample submission (personal communication, 2012), an average of 364 urine samples (median 257, range 25 to 1,162) per year per eligible practice are sent in to the GUH laboratory. Considering that the intervention will run over a six-month period (Phases 2 and 3) and without any adjustment for the total number of UTI-related consultations, an estimated 3,640 urine samples will be submitted from 20 participating practices during this period. If 56% of these patients are prescribed an antimicrobial over a six-month period, a total of 2,038 patients will be prescribed an antimicrobial.

#### Secondary outcome: decrease in the quantity of consumption (control - intervention arm B)

To estimate the potential power to detect this secondary outcome, an additional sample size calculation was carried out.

• 56% of the patients presenting with a UTI, and for whom a urine sample was submitted for microbiological analysis, received a prescription [8].

• A 20% decrease in consumption can be achieved through delayed prescribing [42].

Taking account of the cluster effect and based on previous ICC estimates, a total sample size of 240 patients recruited from 20 practices gives a power of 80% to detect a significant change in the proportion of patients who reduce antimicrobial consumption through delayed prescribing if the ICC is 1%.

### Intervention

#### Overview of SIMPle intervention

The SIMPle intervention incorporates components that address GP and patient behaviours relating to antimicrobial prescribing and consumption. Each component is discussed individually. Table 1 provides an overview of all intervention components.

**Table 1:** Overview of SIMPle intervention components

**Intervention component**	**Study arm**	**Description**	**Primary objectives**	**Secondary objectives**
**GP focused**				
Coding workshop	All arms	Demonstrate the purpose of electronically coding UTI consultations using coding guidelines (International Classification of Primary Care code U71) and how to implement uniform coding in routine practice.	Promote routine uniform consultation coding for UTIs.	Gather baseline practice data and knowledge and attitudinal data (questionnaires).
Interactive workshop A	Intervention arm A	Raise the profile of GPs’ antimicrobial prescribing behaviours and their role in the broader issue of AMR.	Encourage GPs to become more aware of their antimicrobial prescribing behaviours through discussion of their role in the development of AMR.	Process evaluation
		Provide guidance on antimicrobial prescribing through adherence to guideline for UTI.		
Interactive workshop B	Intervention arm B	In addition to the core components of interactive workshop A, the recommendation to delay antimicrobial prescribing for UTIs facilitated through shared decision making will be promoted.		Encourage the use of delayed prescribing for UTIs.
				Process evaluation.
Electronic audit and feedback reports	Intervention arms A and B	A monthly report will be available (electronically) to practices summarizing their antimicrobial prescribing patterns for UTIs and antimicrobial resistance patterns of *E. coli*.	To provide a comparison of antimicrobial prescribing for UTIs between practices.	To provide an overview of antimicrobial resistance patterns in *E. coli* causing UTIs.
Stakeholder awareness		Information about the intervention and the issue of AMR will be provided to other stakeholders; pharmacists, practice receptionists and practice nurses.	To update all stakeholders on the overall aim of the intervention.	
Computer “prompt” application	Intervention arm A	Upon entry of the uniform UTI code, automated electronic prompts will appear on the GPs computer screen.	Provide the recommended antimicrobial treatment for a UTIs.	Remind the GP to collect urine samples and patients’ mobile telephone numbers.
	Intervention arm B		Provide the recommended antimicrobial treatment for a UTI and suggest delayed prescribing.	
**Patient focused components**				
Informational leaflets	Intervention arms A and B	Leaflets will outline the aim of the SIMPle study and highlight issues relating to consuming antibiotics. Intervention arm B patients will receive additional information, outlining the benefits of delayed prescribing for UTIs.	To increase awareness of the side effects, resistance and lack of the availability of new antibiotics.	To inform the patient of the study. To support the use of delayed prescribing for UTIs (patients in intervention group B only).
Educational infomercial		An educational video will be displayed in practice waiting rooms. The video will outline the key issues associated with AMR to patients.		
**GP and patient focused components**				
Website	Intervention arms A and B	The website will provide more information on the issues of AMR.	To provide access to an online survey.	Provide background to the SIMPle study.

### GP components

#### Intervention workshops

A coding workshop will be delivered to all practices at the beginning of Phase 1. Routine coding for UTIs using the International Classification of Primary Care code (U71: 'cystitis, urinary infection, other’) will be demonstrated. The advantage of routine coding of UTI consultations will be to facilitate the generation of electronic audit and feedback reports, which will be extracted through the iPCRN system (see further).

Contact information for GPs (for example, mobile telephone numbers, email addresses) will also be collected to facilitate reminders to code throughout the study period.

Phase 2 will begin with an interactive workshop for intervention arms A and B. The interactive workshops promote changes (where necessary) in antimicrobial prescribing for the treatment of UTIs by presenting an overview of prescribing and AMR, discussing the role of the GP in the spread of AMR and the potential positive impact of prescribing according to guidelines.

In addition, the interactive workshop delivered to practices in intervention arm B will support the GPs in recommending delaying antimicrobial treatment by 48 hours where appropriate. Based on the information provided in the workshop, the GP can discuss the benefits of delayed antimicrobial treatment for UTIs with the patient. This two-way communication will encourage patients to make an informed decision on their treatment. The decision to delay treatment will be shared between the GP and the patient.

The workshops will be delivered to each practice by members of the research team. General practitioners attending the workshops can receive continued professional development recognition for their participation from the Irish College of General Practitioners.

#### Computer prompt application

For intervention arms A and B, a computer prompt has been developed for use within the selected GP practice management software system. This prompt summarizes the recommendations for first-line antimicrobial treatment and will appear on the computer screen when the GP enters the International Classification of Primary Care code (U71) for 'cystitis, urinary infection, other’. In addition, for practices in intervention arm B, the prompt will include a recommendation to consider delayed antimicrobial prescribing. In intervention arms A and B this prompt will also remind the GP to collect patients’ mobile telephone numbers.

#### Electronic audit and feedback reports

Electronic audit and feedback reports will be available to download by GPs through the iPCRN. These reports will provide the practice with information on antimicrobial prescribing for UTI in comparison with the aggregated information from the other practices participating in the intervention. AMR patterns of bacteria causing UTIs will also be presented to the practice. The audit and feedback reports will be available to intervention arms A and B from the start of Phase 2, and to the control arm at the end of Phase 3.

#### Stakeholder awareness

All relevant gatekeepers who are directly or indirectly involved in the implementation of the intervention will receive information on the SIMPle study. These gatekeepers include practice staff, such as the receptionist, practice managers and nurses. Local pharmacists will also be informed of the intervention prior to its launch. The use of delayed antimicrobial prescribing for UTIs will be communicated to local pharmacists to provide support in the event of a patient requiring further information.

#### Patient components

The key messages about antimicrobials underlying the design of all patient focused materials are:

1. Taking an antimicrobial for an infection now increases your chances of having a resistant bacterial infection in the future.

2. Owing to the development of AMR, we are running out of antimicrobial treatment options for more serious bacterial infections.

#### Informational leaflets

Patients presenting with symptoms of a UTI to their GP will receive an information leaflet describing the study, the purpose of the collection of their mobile phone numbers, facts about antimicrobials and the issues surrounding the development of AMR in bacteria. Patients visiting practices from intervention arm B will receive the leaflet with additional information on delayed prescribing of antimicrobials as a potential treatment option for UTIs.

#### Patient education

An infomercial (information commercial) explaining the two key messages (as previously outlined) has been developed for display in practice waiting rooms. An infomercial is a short video representation of information, data or knowledge intended to present complex information quickly and clearly. The infomercial will be displayed in an enclosed and fixed iPad in the practice waiting room. There will also be an option to play an AMR-themed game on this iPad, which will contain the same two messages as stated above. All intervention practices will be offered an iPad for their waiting room at the start of Phase 3 (Figure 1). The impact of this component on antimicrobial prescribing and consumption will be evaluated in comparison with Phase 2. Both the infomercial and the game will be made freely available for download at the end of Phase 4.

#### The control arm

To gather comparable data on UTI consultations, practices in the control arm will complete a coding workshop at the beginning of Phase 1. For the remainder of the intervention, the control arm will provide 'usual care’ and will receive their audit and feedback reports at the end of intervention Phase 3. Practices in the control arm will also submit urine samples from UTI patients and request patients’ mobile telephone numbers for the duration of the intervention.

#### Ethical approval

Ethical approval including a patient opt-out methodology was obtained from the Irish College of General Practitioners.

#### Informed consent

Informed consent for practice participation in the intervention and for the remote extraction of anonymized UTI patient consultation data through the iPCRN will be obtained from the practices at the start of the coding workshop in Phase 1. Each practice will also receive posters to display in their waiting room to inform patients of the practice’s decision to participate in the SIMPle study. The GP will request the patient’s mobile telephone number and outline the aim of the study during the UTI consultation. Patients will be required to confirm their consent by replying to the first text message they receive before continuing with the text-messaging process.

### Quantitative data collection

Data collection will involve five quantitative components: consultation data obtained through the iPCRN; questionnaire data from the GPs, along with a practice profile from the practice manager; Health Service Executive Primary Care Reimbursement Services (HSE-PCRS) prescribing data; and patient follow-up information through text messages. Data for the GPs will be collected at the beginning of Phase 1, immediately after the patient education intervention (Phase 3) and after a six-month follow-up (Phase 4). The collection of patient data will continue throughout Phases 2 and 3.

#### Consultation data

For the purpose of the intervention, UTI consultation data will be extracted remotely through the iPCRN. Extracted anonymized consultation information will be comprehensive and will include, at a practice level:

• Total number of consultations per month;

• Total number of consultations for UTIs per month (coded U71);

• Practice information (GPs, practice nurse, dispensary);

• Number of times an audit was generated (to monitor coding).

For the patients, the following information will be collected:

• Consultations (numbers, dates);

• UTI consultations (dates);

• Urine sample results (date, outcome, resistance profile);

• Antimicrobial prescribing (type, date, dosage);

• Other prescribed medication (type, date, dosage);

• Demographic data including age, sex, medical card status and comorbidities (if coded).

#### Questionnaires

Additional information collection will take place at primary points throughout the intervention, including the administration of a questionnaire to collect data on knowledge and attitudes towards antimicrobial prescribing by GPs. This questionnaire will be administered to GPs at the beginning of Phase 1, at the coding workshop and at the end of Phase 3, to measure potential changes in knowledge, attitudes and behaviours of GPs in each arm. A practice profile will be completed at the beginning of Phase 1, to determine number of whole-time equivalent GPs and practice size.

#### Health Service Executive Primary Care Reimbursement Services

The HSE-PCRS provides free health services under the General Medical Services scheme. Approximately 30% of the Irish population is entitled to free medical care, including medication under this scheme. Regional prescribing data from practices in the study will be compared with HSE-PCRS data [70].

#### Follow-up data

Evaluative information from the patients will be obtained via text message. These text messages will require a minimal response (at no cost) from the patient and will be used to assess actual consumption of the prescribed antimicrobial. Patients can discontinue receiving these text messages at any time by replying STOP.

#### Online survey

After clearance of symptoms, assessed via text messaging, patients who were followed up will be invited to complete a brief online survey. This survey includes questions regarding the patient’s attitudes to antimicrobials, consumption of antimicrobials and awareness of the potential implications of AMR. Patients will be incentivized to complete the online survey.

### Quantitative data analysis

Data comparisons will be made between the start and end of the intervention. To assess sustainability, an additional comparison will be made six months after the end of the intervention. An overview of the data collected through the iPCRN, as outlined in data collection, will be presented for both practice and patient variables. Data will be presented and compared between each arm. Patients’ characteristics will be compared between the group who were followed up by text messaging and the group who did not participate in text messaging.

#### Primary outcome: adherence to the guidelines for antimicrobial prescribing in primary care in Ireland

Comparison of antimicrobial prescription data will be made between control and intervention arm A.

• Each consultation will be categorized according to whether an antimicrobial was prescribed (yes/no) and if so, whether first-line antimicrobials were prescribed (yes/no).

• Antimicrobial prescribing rates will be determined for each intervention arm per month for first-line, second-line and other antimicrobials as specified in the Guidelines for Antimicrobial Prescribing in Primary Care in Ireland.

• The proportion of antimicrobial prescribing according to first-line treatment for each intervention arm will be calculated by determining the number of first-line prescriptions as a percentage of the total number of antimicrobial prescriptions for UTIs.

#### Outcome: frequency of antimicrobial prescribing and consumption

The frequency of antimicrobial prescribing will be compared between the control and each intervention arm (arms A and B).

• Consultations will be analyzed according to whether an antimicrobial was prescribed or not (yes/no).

• The prescription rates per group per month for each antimicrobial group will be calculated as the number of antimicrobial prescriptions per 1,000 consultations per month.

• Comparison of the overall reduction over the period of the intervention will be based on the percentage of consultations where an antimicrobial was prescribed at the start of the intervention, as compared with the end.

The frequency of antimicrobial consumption will be compared between intervention arms A and B, according to their response to the text messages.

#### Outcome: the effect of delayed prescribing

The frequency of delayed prescribing measured as a percentage of overall prescribing for UTIs will be compared between the intervention arm B and the control. Compliance with delayed prescriptions will be measured by the percentage of patients who waited the recommended 48 hours (information obtained by text message).

#### Outcome: reconsultation visits

Reconsultation will be determined by the number of reconsultation UTI visits to the GP within one month of initial treatment.

##### Outcome: change in cognitive beliefs (knowledge and attitudes)

Change in cognitive beliefs at the start of Phase 1 to the end of Phase 3 will be assessed by comparing the percentage change in cognitive beliefs (knowledge and attitudes) relating to antimicrobial prescribing behaviour between the three arms.

#### Outcome: impact of patient educational material

A potential additional impact of patient education will be evaluated by comparing prescribing and consumption between A1 and B2 (no support) and A2 and B1 (support) (see Figure 1).

### Method of quantitative data analysis

Quantitative analysis will be undertaken using SPSS for Windows release 20.0 (SPSS Inc., Chicago, IL, USA) and Stata 11 (Stata Corporation, Texas). Multilevel analysis will be performed using MLwiN version 2.21 software and cost-effectiveness analysis will be conducted using Excel and Stata 11. Statistical significance will be set at the 5% level for all outcomes.

Multivariate analysis will examine intervention and control arm changes from baseline measures and will follow the principle of intention to treat. Outcomes will be compared between intervention (arms A and B) and the control arm, using a multilevel logistic modelling approach to control for baseline prescribing as well as other confounding variables at patient and practice level. All statistical analysis will be undertaken in accordance with current guidelines for randomized controlled trials [71].

### Qualitative data collection

Qualitative data will be used to assist in the process evaluation of the intervention.

Data will be collected through semistructured interviews with GPs, practice staff and patients from intervention and control practices at the end of the study period. GPs and practice staff will be randomly selected from participating practices (*n* ≈ 15 to 40). Patients can opt in to participate by providing their contact details at the end of the online patient survey.

The intervention will be evaluated from the GP’s perspective:

• Changes in GPs’ attitudes towards antimicrobial prescribing;

• How the intervention is implemented;

• How the intervention is integrated with other practice activities;

• How well GPs understand the intervention;

• Whether elements of the intervention are particularly important or problematic.

The intervention will be evaluated from the patient’s perspective:

• Satisfaction with the GP consultation;

• The process of care;

• Evaluate the intervention components as stated above.

### Qualitative data analysis

Analysis of qualitative data will be completed using NVivo-9 qualitative data analysis software. Data analysis will follow an inductive approach through thematic analysis. A cross-comparison of qualitative analysis will be conducted by two team members, one not directly involved in the interview process; along with active discussion of findings with the wider project team, this will help ensure the rigour of the thematic analysis.

### Economic evaluation

The economic cost of UTIs in primary care consists of direct costs associated with health care utilization and drug costs, indirect costs associated with lost productivity, premature mortality and intangible costs, such as the cost of pain and discomfort to the individual. In addition, increasing resistance to antimicrobials has implications for the economic costs of UTIs. In particular, potential treatment failure as a result of resistance means an increase in the use of more expensive alternative treatments, as well as potential increases in healthcare utilization.

The economic analysis will take a two-staged approach.

1. A within-intervention analysis will be conducted, using the cost per rate of first-line antimicrobial prescribing to assess the costs and effects of the intervention arms in comparison with the control arm.

2. Modelling analysis of the impact of prescribing adherence will be undertaken. This will facilitate the identification, valuation and extrapolation of the wider impact of prescribing adherence.

The costs taken into account will be:

• Intervention costs: direct costs will include intervention costs, capital costs, cost of providing workshops, learning materials, leaflets and any other materials.

• Health services costs include GP consultation time, use of hospital services, additional laboratory service costs and prescription costs.

The cost-effectiveness will be assessed by relating the mean differential cost per practice between the intervention arms and the control, to their differential effectiveness. Incremental cost-effectiveness ratios will be calculated and subjected to sensitivity analysis. The primary outcome will be cost per prescription of first-line antimicrobials.

In addition, it is likely that some of the costs and consequences of the intervention will continue after the intervention period has ended, for which an additional economic evaluation will be performed. This modelling analysis will facilitate the extrapolation of the costs and outcomes over time with data obtained from the within-intervention analysis. Where there are evident gaps in the available data, data will be obtained from the literature following a systematic approach.

## Discussion

### Anticipated outcomes

Utilizing a multidisciplinary perspective and social marketing conceptual framework, this study will contribute an understanding of the key behaviours and social norms that motivate change in GPs antimicrobial prescribing and patient antimicrobial consumption behaviours. The intervention will provide an evaluation of the different parts and thereby provide evidence to implement individual or all parts of the intervention beyond the study area and period.

### Anticipated publications

• A qualitative study of the formative research that underpinned the development of the intervention.

• A description of the multidisciplinary intervention to improve quality and quantity of antimicrobial prescribing in primary care (protocol).

• A quantitative analysis of the impact of the intervention on the quality and quantity of antimicrobial prescribing in primary care.

• An economic analysis of the intervention to improve antimicrobial prescribing in primary care including future cost estimates.

• Given the community focus of this research, findings will be disseminated through local print and online media, to include the widest possible audience.

### Limitations of the study

The method of data collection used requires a coding workshop to improve routine consultation coding for UTIs in all practices participating in the intervention, including those in the control group. The inclusion of the control group in the coding workshop may affect the outcome; however antimicrobial treatment was not discussed at these workshops. To limit this bias, researchers delivering the workshops were blinded as to randomization of practices to intervention and control. This bias is anticipated to increase awareness in the control group, resulting in a potentially smaller difference between intervention and control arms.

Computerized remote data extraction will be used to assess differences between the intervention and control arms. Data analysis will not be blinded. However, as the outcome is an absolute comparison in first-line prescriptions of antibiotics, before and after the intervention, the risk of bias is minimal.

The use of patient mobile telephone numbers for evaluation purposes, while novel and direct, introduces selection bias, owing to different levels of mobile phone penetration and familiarization with their use. For research evaluation purposes, however, the implementation of follow-up by mobile telephone technology allows evaluation of consumption (and thereby the impact of delayed prescribing) rather than simply the evaluation of prescribing behaviour by GPs. However, since the sample can be related back to the source population and the selection bias can be estimated, the final evaluation can be considered within this context.

## Trial status

The intervention was registered with ClinicalTrials.gov on 26 July 2013, ID number NCT01913860. At the time of protocol submission, geographical areas and practices have been identified and contacted, and 30 practices have been recruited.

## Abbreviations

AMR: Antimicrobial resistance; GP: General practitioner; GUH: Galway University Hospitals; HSE-PCR: Health service executive primary care reimbursement services; ICC: Intraclass correlation coefficient; iPCRN: Irish primary care research network; SIMPle: Supporting the improvement and management of prescribing for urinary tract infections; UTI: Urinary tract infection.

## Competing interests

The authors declare that they have no competing interests.

## Authors’ contributions

AV, AWM, CD, MC, EOS, KB and MOD conceived the study and coordinated the application for funding. SD, CD and AC developed the formative framework, AV developed the quantitative framework. AC and EOS developed the economic analysis. AC, SD, SG and AV drafted sections of the manuscript. All authors read and approved the final version of the manuscript.
